# Reduced pro-inflammatory profile of γδT cells in pregnant patients with rheumatoid arthritis

**DOI:** 10.1186/s13075-016-0925-1

**Published:** 2016-01-22

**Authors:** Manuela Tham, Gabriele R. Schlör, Daniel Yerly, Christina Mueller, Daniel Surbek, Peter M. Villiger, Frauke Förger

**Affiliations:** Department of Rheumatology, Immunology and Allergology, University Hospital and University of Bern, Bern, Switzerland; Graduate School, University of Bern, Bern, Switzerland; Department of Obstetrics and Gynecology, University Hospital and University of Bern, Bern, Switzerland

**Keywords:** Pregnancy, rheumatoid arthritis, ankylosing spondylitis, disease improvement, gammadelta T cells

## Abstract

**Background:**

During pregnancy, many patients with rheumatoid arthritis (RA) experience disease improvement, whereas patients with ankylosing spondylitis often suffer from persistent active disease. Here we investigated whether pregnancy-related changes in disease activity were associated with changes in the proportion and function of γδT cells.

**Methods:**

The study population comprised 55 patients with RA, 31 patients with ankylosing spondylitis, and 35 healthy controls. Among these participants, 28 RA patients, 21 ankylosing spondylitis patients, and 23 healthy controls were investigated once before conception when possible, at each trimester of pregnancy, and at 8 weeks postpartum. Data were compared with age-matched non-pregnant patients to obtain disease-related background. In all subjects, peripheral Vδ1 and Vδ2 T cells were analyzed for cell frequencies, the activation marker CD69, the cytotoxicity markers NKG2D and NKG2A, and the intracellular cytokines tumor necrosis factor (TNF)α, interferon (IFN)γ, interleukin (IL)-17 and IL-10.

**Results:**

Pregnant patients showed a decreased Vδ2/Vδ1 ratio in the third trimester, which resulted from a slightly reduced proportion of Vδ2 cells. Changes in RA disease activity during pregnancy and postpartum were not associated with numerical proportions of γδT cells but with changes of the cell activation marker CD69 on Vδ1 and Vδ2 cells. Only RA patients showed reduced proportions of TNFα-positive Vδ1and Vδ2 cells and IFNγ-positive Vδ2 cells at the third trimester of pregnancy, a finding that was not apparent in the entire population of CD3 T cells. The proportions of IL-17-positive γδT cells and IL-10-positive γδT cells did not differ between pregnant and non-pregnant women of the different groups.

**Conclusions:**

Changes of disease activity in pregnant RA patients were associated with functional changes in both γδT cell subsets. This reduced pro-inflammatory profile of γδT cells might contribute to the immunomodulation resulting in pregnancy-induced improvement of RA.

**Electronic supplementary material:**

The online version of this article (doi:10.1186/s13075-016-0925-1) contains supplementary material, which is available to authorized users.

## Background

Successful pregnancy requires an extraordinary state of natural immunomodulation to permit tolerance towards the haploidentical fetus [1]. The maternal immune system is altered to allow tolerance of fetal antigens while still being able to fight against infections. These immunomodulatory effects of pregnancy have varying influences on autoimmune rheumatic diseases. Rheumatoid arthritis (RA) improves in the majority of patients, whereas ankylosing spondylitis (AS) often remains active or is aggravated [2]. The understanding of relevant factors involved in the pregnancy-induced improvement of RA may enlarge our understanding of pathogenic factors involved in RA.

In this context, γδT cells are of interest since they display immunoregulatory features during pregnancy [3]. These cells are unconventional CD3^+^ T cells that show features of both the innate and the adaptive immune system [4]. In contrast to αβT cells (e.g., CD4 or CD8 T cells), γδT cells recognize non-peptide antigens independently of classical major histocompatibility complex (MHC) molecules [4]. γδT cells exert their main functions through cytokine release, cytotoxicity, or antigen presentation [4]. In the peripheral blood of healthy humans, about 5 % of lymphocytes express γδT cell receptors [5]. Two main subsets of γδT cells bear different variable delta (Vδ) chains, Vδ1 and Vδ2, and show distinct tissue distributions and functions.

At the fetomaternal interface, γδT cells recognize fetus-derived trophoblast cells that do not express polymorphic classical MHC class I molecules [6]. The pregnant uterus shows an increased number of γδT cells [7]. Vδ1 T cells are the predominant subset in the decidua, where they produce interleukin (IL)-10 and transforming growth factor beta [8], while Vδ2 T cells predominate in the peripheral blood of healthy pregnant women [9, 10].

In autoimmune diseases, γδT cells have shown both pro- and anti-inflammatory responses. In the collagen-induced arthritis mouse model of RA, γδT cells function differently at different disease stages, playing a pro-inflammatory role at arthritis onset and an anti-inflammatory role once the disease is established [11]. Only very limited knowledge is available about γδT cells in RA or AS in humans. One study reported reduced Vδ2 T cells in the peripheral blood of RA patients compared to that of healthy controls [12]. Both Vδ1 and Vδ2 have been found in the synovium of RA patients with a predominance of interferon (IFN)γ-producing Vδ1 cells [13, 14].

It is currently unknown whether γδT cells play an immunoregulatory role in pregnancy-related amelioration of RA. We hypothesized that pregnancy has a systemic tolerance-inducing effect that would shift the functional plasticity of circulating γδT cells towards an anti-inflammatory profile, potentially impacting disease activity in RA. This shift would not be observed in a disease that remains active during pregnancy, such as AS. In the present study, we investigated phenotypical, and cytokine changes of circulating γδT cells during pregnancy in patients with RA as compared to those with AS and healthy controls.

## Methods

### Patients

Here we prospectively studied 72 pregnant women: 28 with RA, 21 with AS, and 23 healthy controls. Pregnant individuals were examined once before conception when possible (up to 6 months before conception), once at each trimester (during gestational weeks 10–12, 20–22, and 30–32), and once at 8 weeks postpartum. Additionally, we studied 49 non-pregnant age-matched individuals, including 27 women with RA, 10 with AS, and 12 healthy women. Table 1 shows the patient characteristics and medications. All patients gave written informed consent, and the study was approved by the ethical committee of the Canton of Bern.

**Table 1:** Patient characteristics

	Healthy control	Rheumatoid arthritis	Ankylosing spondylitis
Total number			
Total	35	55	31
Non-pregnant	12	27	10
Pregnant	23	28	21
Age in years; median (range)			
Non-pregnant	30.5 (24–48)	32.5 (17–51)	35 (30–47)
Pregnant	33 (23–41)	32 (28–39)	32.5 (27–39)
Rheumatoid factor positive			
Non-pregnant		19 (59.4)	
Pregnant		13 (46.4)	
ACPA positive			
Non-pregnant		17 (53.1)	
Pregnant		12 (42.9)	
HLA-B27 positive			
Non-pregnant			7 (70)
Pregnant			14 (66.7)
Medication			
NSAID			
Non-pregnant	–	25 (75.8)	7 (70)
3^rd^ trimester	–	4 (14.3)	5 (23.8)
Postpartum*	–	7 (25)	9 (42.9)
Prednisone**			
Non-pregnant	–	12 (36.4)	1 (10)
3^rd^ trimester	–	13 (46.4)	3 (14.3)
Postpartum*	–	9 (32.1)	2 (9.5)
Sulfasalazine			
Non-pregnant	–	8 (24.2)	1 (10)
3^rd^ trimester	–	5 (17.9)	–
Postpartum*	–	7 (25)	1 (4.8)
Antimalarials			
Non-pregnant	–	1 (3)	–
3^rd^ trimester	–	2 (7.1)	–
Postpartum*	–	5 (17.9)	–
Methotrexate			
Non-pregnant	–	7 (21.2)	–
3^rd^ trimester	–	–	–
Postpartum*	–	–	–
Leflunomide			
Non-pregnant	–	1 (3)	–
3^rd^ trimester	–	–	–
Postpartum*	–	–	–
TNF inhibitors			
Non-pregnant	–	5 (15.2)	3 (30)
3^rd^ trimester	–	–	–
Postpartum*		–	2 (9.5)

All values are shown as *n* (%) except where indicated otherwise. *Postpartum: 6–8 weeks after birth; ** < 15 mg/day. *ACPA* anti citrullinated peptide antibodies, *NSAID* Non-steroidal anti-inflammatory drug (until gestation week 32), *TNF* Tumor necrosis factor

All patients were recruited from the pregnancy clinic of the Department of Rheumatology, Immunology, and Allergology and the Department of Obstetrics and Gynecology at the Inselspital of Bern, Switzerland. RA patients fulfilled the American College of Rheumatology criteria [15]. AS patients all had established axial involvement and fulfilled the modified New York Criteria [16]. RA disease activity was measured using the Disease Activity Score 28–C-reactive protein (DAS28-CRP) with three variables: swollen joint count, tender joint count, and C-reactive protein (CRP). AS disease activity was measured using the Ankylosing Spondylitis Disease Activity Score–C-reactive Protein (ASDAS-CRP). Serum CRP was measured either by Nycocard CRP Single Assay (Alere GmbH, Wädenswill, Switzerland) or high-sensitivity CRP test (Department of Clinical Chemistry, Inselspital, University of Bern, Switzerland). The healthy control individuals included in the study each had a CRP below 5 mg/L. Patients and healthy women with infections were excluded from the study.

### Cell preparation and flow cytometric analysis

Peripheral blood mononuclear cells (PBMCs) were isolated from heparinized blood by standard density-gradient centrifugation over Biocoll (Biochrom AG, Berlin, Germany). For the frequency analysis of CD3, Vδ1 and Vδ2, and for the analysis of the activation marker and the cytotoxicity marker, the following directly labeled monoclonal antibodies were used: the PerCP-conjugated antibody CD3 (clone SK7) from Biolegend, the fluorescein-isothiocyanate-conjugated antibodies Vδ1 (clone TS-1) and Vδ2 (clone B6) from Thermo Scientific (Waltham, MA, USA), the phycoerythrin-coupled antibodies CD69 (clone FN50, Biolegend, San Diego, CA, USA), NKG2A (clone Z199, Beckman Coulter, Brea, CA, USA), and the allophycocyanin-coupled antibodies NKG2D (clone 1D11). Immunofluorescence staining was performed after washing the cells twice with phosphate-buffered saline containing 1 % human serum. Cells were incubated for 20 minutes with each monoclonal antibody.

For the intracellular cytokine staining, cells were plated in 48-well plates at 1 × 10^6^ cells/100 μL in complete RPMI 1640 containing 1× non-essential amino acids, 1× glutamine, 1× sodium pyruvate, 1× kanamycin (Life Technologies, Carlsbad, CA, USA), and 5 % pooled human serum (Blood Transfusion Service, Bern, Switzerland) and stimulated with phorbol myristate acetate (PMA; 25 ng/mL; Sigma Aldrich, St. Louis, MO, USA) and ionomycin (1 μg/mL; Sigma Aldrich) for 4 hours in the presence of the protein transport inhibitor Brefeldin A (10 μg/mL; Sigma Aldrich). Intracellular cytokine staining was performed with the following antibodies: phycoerythrin-coupled antibodies, tumor necrosis factor (TNF)α (clone MAb11) and IL-10 (clone JES3-19 F1) from BD Biosciences (San Jose, CA, USA), and allophycocyanin-coupled antibodies, IFNγ (clone B27) from Biolegend and IL-17A (clone eBIO64DEC17) from eBioscience (San Diego, CA, USA). After surface and intracellular staining of PBMCs, data acquisition was performed using FACSCalibur 4-Color Cytometer (BD Biosciences), and data were analyzed using FlowJo Software (FlowJo, Ashland, OR, USA). Intracellular cytokine data were studied using both the proportion of cytokine-positive cells and the geometric mean fluorescence intensity (MFI) of a gated cell population.

### Statistical analysis

All data are reported as the median and range. The Mann-Whitney U test was used for unpaired data analysis and group-wise comparison. The Wilcoxon signed-rank test was applied for longitudinal comparisons of paired samples. Linear regression analysis was used to investigate the association between disease activity and γδT cell features. Data were analyzed using IBM SPSS Statistics 21 software. A *P-*value below 0.05 was considered significant.

## Results

### Disease activity improved in pregnant RA patients

**Fig. 1:**
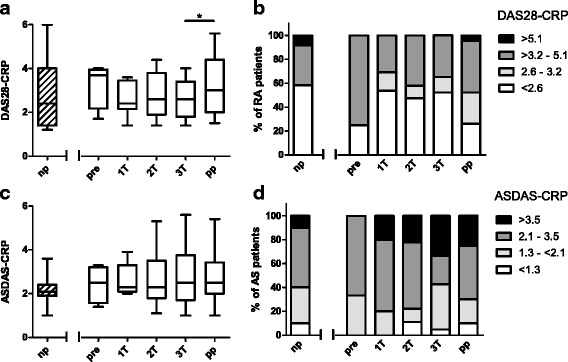
Disease activity before, during and after pregnancy. **a** Disease activity measured by the Disease Activity Score in 28 joints and C-reactive protein (*DAS28-CRP*) for 28 longitudinally followed pregnant rheumatoid arthritis (*RA*) patients and for 27 non-pregnant RA patients. **b** The graph shows the percentages of the abovementioned patients grouped according to DAS28-CRP scores into: remission (DAS28-CRP <2.6), low disease activity (DAS 28-CRP 2.6–3.2), moderate disease activity (DAS28-CRP >3.2–5.1) and high disease activity (DAS28-CRP >5.1). **c** Disease activity measured by the Ankylosing Spondylitis Disease Activity Score and C-reactive protein (*ASDAS-CRP*) for 21 longitudinally followed pregnant ankylosing spondylitis (*AS*) patients and for 10 non-pregnant (*np*) AS patients. **d** The graph shows the percentages of patients grouped according to ASDAS-CRP scores into: inactive disease (ASDAS <1.3), moderate disease activity (ASDAS 1.3– < 2.1), high disease activity (ASDAS 2.1–3.5) and very high disease activity (ASDAS >3.5). Pregnant patients were analyzed once before pregnancy (*pre*), at each trimester (*1T*, *2T*, *3T*) and 8 weeks postpartum (*pp*). Boxplots show the median and the interquartile ranges. *P < 0.05

A total of 49 pregnant patients and 23 healthy pregnant women were examined before (when possible), during and after pregnancy (Table 1). In RA patients, the disease activity scores according to DAS28-CRP were lower at the third trimester than 8 weeks postpartum (*P* = 0.03; Fig. 1a). Though less medication was used by the pregnant patients than by the non-pregnant control group, inactive disease was commonly seen in pregnant RA patients, with 58–69 % of RA patients showing low disease activity (DAS28-CRP scores <3.2; Fig. 1b). In contrast, disease activity in AS patients as measured by ASDAS-CRP remained unchanged during and after pregnancy (Fig. 1c). During pregnancy, 57–80 % of AS patients experienced active disease (ASDAS-CRP scores >2.1; Fig. 1d).

### Discrete quantitative changes of Vδ1 and Vδ2 cells in pregnant patients

**Fig. 2:**
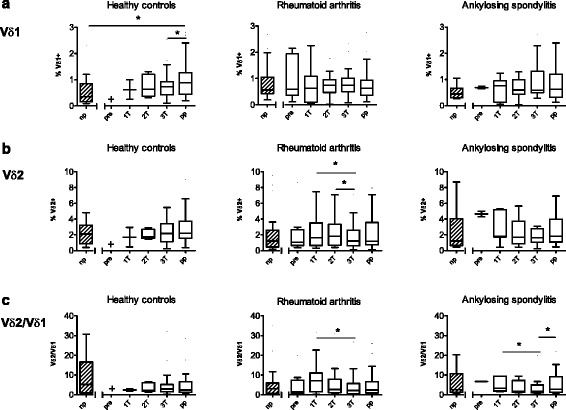
Longitudinal changes of Vδ1 and Vδ2 T cells during and after pregnancy. The proportions of Vδ1 (**a**) and Vδ2 cells (**b**) and the Vδ2/Vδ1 ratio (**c**) are shown for healthy controls (*left panels*), patients with RA (*middle panels*) and patients with AS (*right panels*). Pregnant individuals were studied before pregnancy (*pre*), at each trimester (*1T*, *2T*, *3T*) and postpartum (*pp*). Values were compared with age-matched non-pregnant (*np*) individuals in each group. Frequencies of Vδ1 and Vδ2 T cells are shown as percentages of CD3-positive T cells in isolated PBMCs. Boxplots show the median and the interquartile ranges. + no data available. *P < 0.05

With regard to the percentages of Vδ1 and Vδ2 T cells within the CD3^+^ population, we first investigated the differences during and after pregnancy as well as the differences between pregnant and non-pregnant controls. Because of the lack of sufficient pre-pregnancy data in healthy women and AS patients, pre-pregnancy data were excluded from the analysis. In healthy subjects, the postpartum proportion of Vδ1 T cells was higher compared to that of the third trimester (*P* = 0.02) and higher compared to that of non-pregnant controls (*P* = 0.04; Fig. 2a). Concerning Vδ2 cells of longitudinally followed healthy subjects, their frequency did not change. The proportion of Vδ1 cells and Vδ2 cells in pregnant healthy women was comparable to that of non-pregnant healthy women (Fig. 2a and b).

Among pregnant RA patients, Vδ1 T cell frequencies did not show significant changes during pregnancy. In contrast, Vδ2 T cells significantly decreased from the first trimester to the third trimester (*P* = 0.02), as well as from the second trimester to the third trimester (*P* = 0.02; Fig. 2b). To detect small changes in the balance between Vδ2 and Vδ1 cells, we calculated the Vδ2/Vδ1 ratio (Fig. 2c). In both pregnant RA patients and pregnant AS patients, the Vδ2/Vδ1 ratio decreased from the first trimester to the third trimester (RA, *P* = 0.01; AS, *P* = 0.03). Among pregnant AS patients, the Vδ2/Vδ1 ratio was lower at the third trimester than at the postpartum examination (*P* = 0.04). These changes in the Vδ2/Vδ1 ratio could not be observed in healthy controls.

Overall, only discrete quantitative changes of Vδ1 and Vδ2 T cells were observed during pregnancy, which were more pronounced in cases of RA than in AS. Interestingly, Vδ2 T cells decreased during pregnancy in cases of RA when disease activity was very low. In contrast, non-pregnant RA patients with active disease (DAS28-CRP >3.2) showed a lower frequency of Vδ2 T cells (median 0.80, range 0.16–3.48) compared to non-pregnant RA patients with the inactive disease (median 2.04, range 0.24–9.54; *P* = 0.04; data not shown).

### Association between disease activity and activation marker expressed by γδT cells of RA patients

**Fig. 3:**
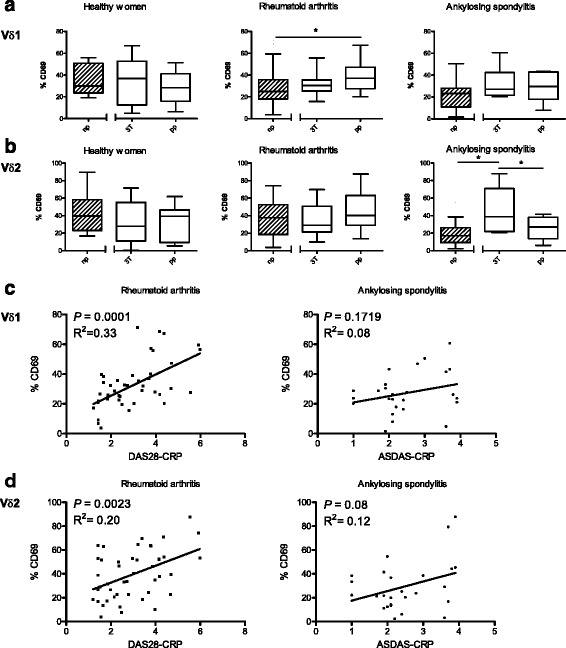
Activation markers expressed by γδT cells and disease activity. Analysis for the proportion of CD69-expressing Vδ1 cells (**a**) and Vδ2 cells (**b**) cells among healthy controls (*left panels*), patients with RA (*middle panels*) and patients with AS (*right panels*). Percentages of CD69-positive cells are compared between non-pregnant (*np*) age-matched women and women studied at the third trimester (*3T*) and postpartum (*pp*). Data were analyzed for correlation between CD69-expressing Vδ1 cells (**c**) and Vδ2 cells (**d**) and the disease activity scores in pregnant (third trimester), postpartum and non-pregnant patients with RA (*left panels*) and AS (*right panels*). Disease activity in RA patients was measured using Disease Activity Score in 28 joints and C-reactive peptide (*DAS28-CRP*) (**a**) and in AS patients using Ankylosing Spondylitis Disease Activity Score and C-reactive peptide (*ASDAS-CRP*) (**b**)

To investigate whether pregnancy-related changes of disease activity were reflected by changes of cell activation, we analysed the expression of the activation marker CD69 on Vδ1 and Vδ2 T cells. The proportion of CD69-positive Vδ1 and Vδ2 cells did not differ between non-pregnant healthy women and those at the third trimester or postpartum time point (Fig. 3a and b). By contrast, RA patients analyzed 8 weeks postpartum showed higher percentages of CD69-positive Vδ1 cells than non-pregnant controls (*P* = 0.01; Fig. 3a). In AS patients, the proportions of CD69-positive Vδ2 were highest at the third trimester and differed from those of non-pregnant AS patients (*P* = 0.02) and from those of postpartum AS patients (*P* = 0.04).The relationship between disease activity and cell activation of γδT cells was then analysed by linear regression analysis in non-pregnant, pregnant (third trimester) and postpartum patients. In RA patients, disease activity as measured by DAS28-CRP correlated with the proportion of CD69-expressing Vδ1 and Vδ2 cells (Fig. 3c). In AS patients, the association of CD69-bearing Vδ2 cells and disease activity measured by ASDAS-CRP showed a slight tendency towards statistical significance (*P* = 0.08; Fig. 3d). There was no significant association between CD69-bearing Vδ1 cells and ASDAS-CRP.

### Increased expression of anti-cytotoxic markers on γδT cells of pregnant RA patients

The cytotoxic response of γδT cells is modulated by the activating natural killer (NK) cell receptor NKG2D and by the inhibiting NK cell receptor NKG2A. We profiled γδT cells for NKG2A and NKG2D expressions to investigate whether pregnancy changed the balance between these functionally different receptors. The proportions of NKG2D-expressing Vδ1 and Vδ2 cells did not change from the first trimester until the postpartum time-point in any group (data not shown). Similar results were seen when analyzing the MFI of NKG2D among the population of Vδ1 and Vδ2 cells.

Pregnant RA patients only displayed a longitudinal change of NKG2A-positive Vδ1 T cells, with an approximately 2.4-fold higher proportion at the second trimester than at the postpartum time-point (median percentage of NKG2A-positive cells: second trimester, 7.53; postpartum, 3.04; *P* = 0.02; data not shown). This difference was not seen when analyzing the MFI of NKG2A among the population of Vδ1 cells. The proportion of NKG2A-expressing Vδ2 cells was stable during and after pregnancy in all groups.

### Reduced TNFα and IFNγ production by γδT cells in pregnant RA patients

To determine the intracellular cytokine profile of γδT cells, freshly isolated PBMCs were activated with PMA and ionomycin in the presence of a Golgi transport inhibitor and stained for TNFα, IFNγ, IL-17 and IL-10. We first analyzed longitudinal changes of cytokine-positive Vδ1 and Vδ2 cells in healthy women and patients. In RA patients, the population of TNFα-producing Vδ1 cells decreased from the second to the third trimester and increased from the third trimester to the postpartum time point (second to third trimester analyzed by percentage of positive cells and by MFI: *P* = 0.04; third trimester to postpartum analyzed by MFI: *P* = 0.04; Additional file 1: Figure S1).

**Fig. 4:**
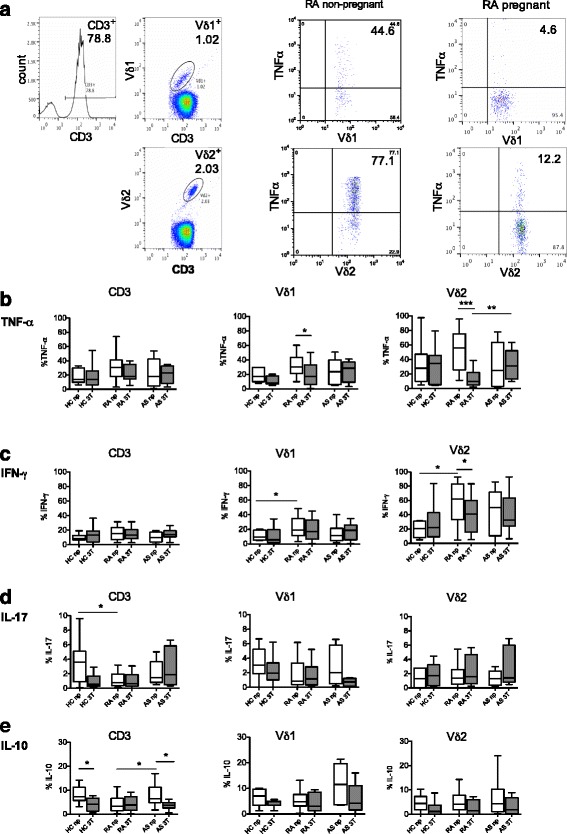
Reduced pro-inflammatory cytokine profile of γδT cells in pregnant versus non-pregnant rheumatoid arthritis patients. PBMCs from healthy women (*HC*), patients with rheumatoid arthritis (*RA*), and patients with ankylosing spondylitis (*AS*) were stained for intracellular cytokines. **a** Representative fluorescence-activated cell sorter analysis plots showing the gating strategy of tumor necrosis factor alpha (*TNFα*)-positive Vδ1 cells and TNFα-positive Vδ2 cells in a non-pregnant and a pregnant RA patient. **b–e** Percentages of CD3 cells, Vδ1 and Vδ2 cells from non-pregnant women (*np*, *white boxes*) were compared to cells from pregnant women (third trimester; *3T*, *grey boxes*) with regard to intracellular production of TNFα (**b**), interferon gamma (*IFNγ*) (**c**), interleukin (*IL*)-17 (**d**) and IL-10 (**e**). *P < 0.05, **P < 0.01, ***P < 0.001

We then compared differences between pregnant and non-pregnant individuals. For pregnant individuals, the third trimester was chosen since this time point was related to clinical changes of disease activity and quantitative changes of γδT cells. Regarding TNFα production, the most pronounced difference appeared between pregnant and non-pregnant RA patients (Fig. 4a and b). Pregnant RA patients showed a lower proportion of TNFα-positive Vδ1 cells compared to non-pregnant RA patients (*P* = 0.04). Similarly, pregnant RA patients showed an approximately 5.7-fold lower proportion of TNFα-positive Vδ2 cells compared to non-pregnant RA patients (*P* = 0.001). With respect to IFNγ (Fig. 4c), non-pregnant RA patients displayed the highest proportions of IFNγ-positive Vδ1 and IFNγ-positive Vδ2 cells, which significantly differed from the median proportions found in non-pregnant healthy controls (Vδ1, *P* = 0.04; Vδ2, *P* = 0.02). Analogous to the TNFα results, the proportion of IFNγ-positive Vδ2 cells was lower in pregnant RA patients compared to non-pregnant RA patients (*P* = 0.03; Fig. 4c). Similar results were found when analyzing the MFI of TNFα and IFNγ among the population of Vδ1 and Vδ2 cells (Additional file 2: Figure S2). Interestingly, the reduced percentages of TNFα- and IFNγ-positive γδT cells observed in pregnant RA patients were not apparent in the entire population of CD3 T cells (Fig. 4b and c, left panels).

With respect to IL-17, we found no significant difference in the proportion of IL-17-positive Vδ1 or Vδ2 cells between pregnant and non-pregnant women (Fig. 4d). The median proportions of IL-17-positive Vδ1 and Vδ2 cells were below 4 %. Regarding IL-10, IL-10-positive cells tended to be lower in pregnant than in non-pregnant individuals for Vδ1 and Vδ2 T cells, an effect which was more pronounced for the population of CD3 cells (Fig. 4e). There was no influence of medication on the proportion of cytokine-positive Vδ1 or Vδ2 cells in non-pregnant patients.

Overall, among all analyzed individuals, only pregnant RA patients showed a reduced production of pro-inflammatory cytokines by Vδ1 and Vδ2 cells.

## Discussion

Pregnancy has a positive effect on RA, but has no beneficial effect on AS. In the present study, we analyzed whether pregnancy-related improvements of RA symptoms were associated with changes of circulating γδT cells. γδT cells are an immune system component that bridge the innate and the adaptive immune response, and they show a functional plasticity ranging from immunoregulatory properties to pro-inflammatory responses. We hypothesized that pregnancy has not only a local- but also a systemic tolerance-inducing effect on immunoregulatory cells such as γδT cells, which might support pregnancy-induced amelioration of RA.

Overall, our results demonstrate a reduced pro-inflammatory cytokine profile of γδ T cells in pregnant patients with RA compared to non-pregnant RA patients. This functional modification of γδT cells occurred in pregnant RA patients of whom 69 % had low disease activity but not in patients with AS, a disease with ongoing disease activity.

Among the two subsets of circulating γδT cells, changes were detected in both the Vδ1 and the Vδ2 cells. Against our expectation based on previous findings on Vδ1 and Vδ2 cells, we did not find an increase in circulating Vδ1 cells during pregnancy that provide a Th2 cytokine pattern as shown for decidual Vδ1 cells [8, 10]. This discrepancy may be explained by the analysis of peripheral Vδ1 and Vδ2 cells and the use of different sampling periods. Among pregnant patients with RA and AS, we identified reduced Vδ2/Vδ1 ratios during the third trimester of pregnancy, which were due to a slight pregnancy-related reduction of Vδ2 cells. However, a previous study also reported reduced Vδ2 cell percentages among non-pregnant RA patients, a finding that was not related to disease activity assessed by CRP and erythrocyte sedimentation rate [17]. By contrast, our cohort of non-pregnant RA patients showed a lower frequency of Vδ2 T cells in cases with active disease (DAS28-CRP >3.2) compared to cases with inactive disease (DAS28-CRP <3.2). Similarly, patients with juvenile idiopathic arthritis (JIA) suffering from more active disease showed lower percentages of synovial Vδ1 and Vδ2 T cells [18]. Therefore, we suppose that Vδ2 T cell frequency variations can be triggered by pregnancy and by disease activity in RA.

However, these quantitative changes may be less important than functional changes of γδT cells in terms of reflecting disease activity. In our cohort of pregnant and non-pregnant RA patients, disease activity was associated with Vδ1 and Vδ2 T cells that were positive for the activation marker CD69. Similarly, high CD69 expression on Vδ2 cells has been reported in inflamed joints of JIA patients [19]. However, in our RA patients, disease activity correlated with CD69 positivity on both Vδ1 and Vδ2 cells, which do not respond to the same antigen. Thus, the increased percentage of CD69-positive Vδ1 and Vδ2 cells might be induced by a disease-related inflammatory environment rather than by specific antigens.

γδT cells exert their effector function via both cytokine release and cytotoxicity. Cytotoxic potency has been described for both Vδ1 and Vδ2 cells [20, 21], along with modulation by the activating NK cell receptor NKG2D or the inhibiting NK cell receptor NKG2A. The placenta secretes NKG2D ligands, which could downregulate NKG2D [22]. Therefore, we expected to find a pregnancy-induced change of the balance between the NKG2D and NKG2A expressions on Vδ1 and Vδ2 cells. However, our results showed that the percentages of NKG2D-expressing Vδ1 and Vδ2 cells remained unchanged during pregnancy and postpartum. In contrast, the percentage of NKG2A-expressing Vδ1 cells was higher during the second trimester than postpartum in RA patients. A previous report demonstrated a pregnancy-related increase of NKG2A-positive γδT cells in healthy women analyzed around gestational week 27 compared to non-pregnant controls [10]. The increased expression of NKG2A on γδT cells might therefore be a pregnancy-related phenomenon. In this respect, one might speculate that trophoblast-derived HLA-E binds to the increased proportion of NKG2A-positive γδT cells, thereby down-modulating their cytotoxic potential [23, 24].

Given that γδT cells can produce the pro-inflammatory cytokines TNFα and IFNγ, which are involved in the pathogenesis of RA and spondyloarthritis [25, 26], it is possible that pregnancy induces some dampening of the TNFα- or IFNγ-producing Vδ1 and Vδ2 cells. This speculation was indeed supported by results in RA patients, but not in AS patients or in healthy controls. Therefore, an effect of pregnancy alone, which would appear in all groups, seems unlikely. However, the local cytokine environment has a decisive role in driving γδT cell function in a way that normal pregnancy gives rise to γδT cells with an anti-inflammatory profile whereas pre-eclampsia gives rise to γδT cells with a pro-inflammatory profile [27, 28]. The anti-inflammatory cytokine milieu created by gestational γδT cells in turn promotes feto–maternal tolerance [28]. Thus, the downregulation of TNFα- and IFNγ-positive γδT cells in pregnant versus non-pregnant RA patients could result from a reduced inflammatory milieu that in turn supports disease improvement.

Disease activity in AS remained unchanged during pregnancy. Accordingly, there was no change of TNFα- and IFNγ-positive γδT cells in pregnant versus non-pregnant AS patients. Previous research in AS has shown that a γδT cell subpopulation bearing the IL-23 receptor is the main source of IL-17-secreting T cells [29]. Our analysis showed similar percentages of IL-17-secreting γδT cells, with no differences between the study groups or between pregnant and non-pregnant individuals. Thus, the persistent active disease present in pregnant AS patients provides a pro-inflammatory milieu that might overrule the regulatory features of γδT cells.

The limitations of our study are the limited and unequal number of patients and controls, and the scarce pre-pregnancy and first trimester data of healthy pregnant women. Differences in the frequency and function of γδT cells might be missed in early pregnancy of healthy controls. However, we assume that age-matched, non-pregnant healthy women would not differ from healthy women examined prior to conception.

## Conclusions

In conclusion, here we observed functional changes of γδT cells that are associated with disease activity in a cohort of pregnant versus non-pregnant RA patients. These functional changes of γδT cells in pregnant RA patients which were mainly reflected by decreases of TNFα- and IFNγ-positive γδT cells seem to be a consequence of an anti-inflammatory environment. No such changes of γδT cells were seen in AS, a disease that remains active during pregnancy. The more tolerogenic profile of γδT cells in pregnant RA patients might contribute to the process of immunomodulation in pregnancy.

## Additional files

Additional file 1: Figure S1. Longitudinal changes of TNFα-producing γδT cells in pregnant RA patients. Patients with rheumatoid arthritis were analyzed for the percentages (**A**) and the mean fluorescence intensities (MFI) (**B**) of TNFα-producing CD3 cells (left panel), Vδ1 cells (middle panel) and Vδ2 cells (right panel) before pregnancy (pre), at each trimester (1T, 2T, 3T) and postpartum (pp). Values are expressed as median and interquartile ranges. **P* < 0.05. (PDF 33 kb) Additional file 2: Figure S2. Reduced pro-inflammatory cytokine profile of γδT cells in pregnant versus non-pregnant RA patients. Peripheral blood mononuclear cells from healthy women (HC), patients with rheumatoid arthritis (RA), and patients with ankylosing spondylitis (AS) were stained for intracellular cytokines. Mean fluorescence intensity (MFI) of TNFα (**A**), IFNγ (**B**), IL-17 (**C**) and IL-10 (**D**) among CD3 cells (left panels), Vδ1 cells (middle panels) and Vδ2 cells (right panels) from non-pregnant (np, white boxes) and pregnant women (third trimester, 3T, grey boxes) were analyzed. Box plots show the median and the interquartile ranges. **P* < 0.05, ***P* < 0.01, ****P* < 0.001. (PDF 55 kb)

## Abbreviations

ACPA: Anti citrullinated peptide antibodies
AS: Ankylosing spondylitis
ASDAD-CRP: Ankylosing Spondylitis Disease Activity Score–C-reactive Protein
CD: Cluster of differentiation
CRP: C-reactive protein
DAS28-CRP: Disease Activity Score 28–C-reactive protein
IFN: Interferon
IL: Interleukin
JIA: Juvenile idiopathic arthritis
MFI: Mean fluorescence intensity
MHC: Major histocompatibility complex
NK: Natural killer
PBMC: Peripheral blood mononuclear cell
PMA: Phorbol myristate acetate
RA: Rheumatoid arthritis
TNF: Tumor necrosis factor
Vδ: Variable delta
