# Supplementary figures and images for: Reduced pro-inflammatory profile of γδT cells in pregnant patients with rheumatoid arthritis
Source: Arthritis Res Ther. 2016 Jan 22;18:26. doi: 10.1186/s13075-016-0925-1 (PMC4722716; doi:10.1186/s13075-016-0925-1)

# Supplementary Figure 1

## Rheumatoid arthritis

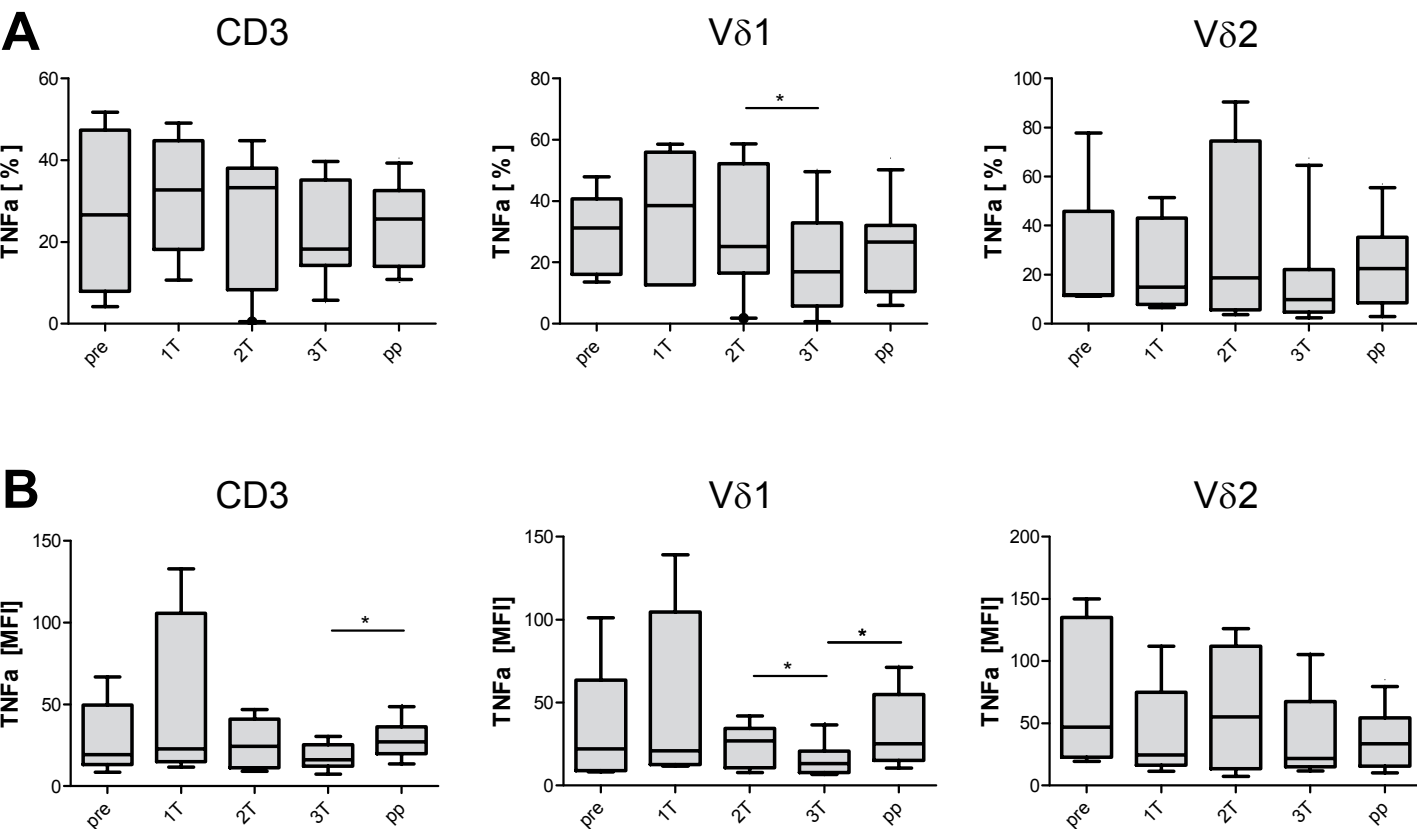

Supplement: Additional file 1: Figure S1. — Longitudinal changes of TNFα-producing γδT cells in pregnant RA patients. Patients with rheumatoid arthritis were analyzed for the percentages (A) and the mean fluorescence intensities (MFI) (B) of TNFα-producing CD3 cells (left panel), Vδ1 cells (middle panel) and Vδ2 cells (right panel) before pregnancy (pre), at each trimester (1T, 2T, 3T) and postpartum (pp). Values are expressed as median and interquartile ranges. *P < 0.05. (PDF 33 kb) [file 13075_2016_925_MOESM1_ESM.pdf]

Supplemental Figure 2

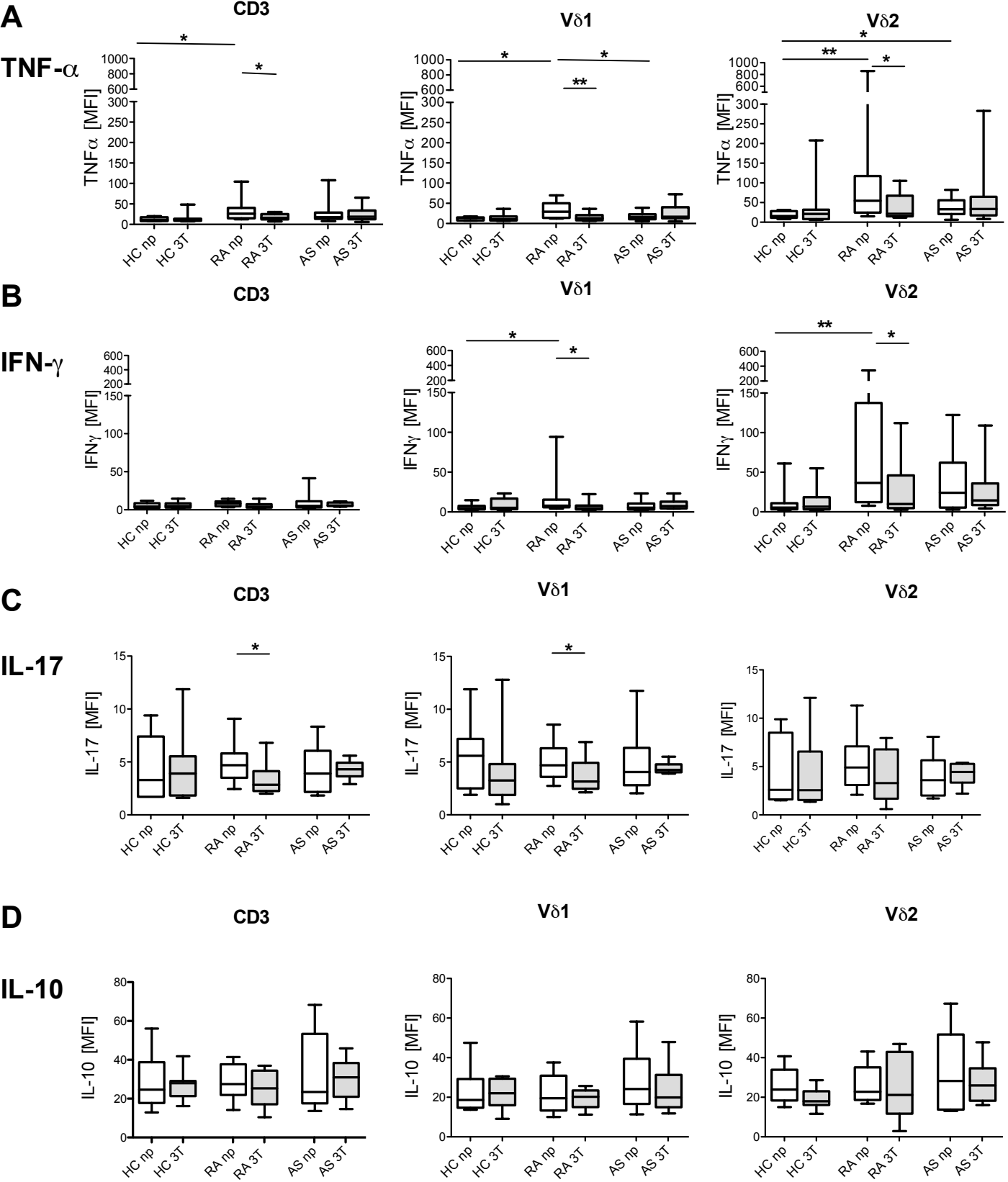

Supplement: Additional file 2: Figure S2. — Reduced pro-inflammatory cytokine profile of γδT cells in pregnant versus non-pregnant RA patients. Peripheral blood mononuclear cells from healthy women (HC), patients with rheumatoid arthritis (RA), and patients with ankylosing spondylitis (AS) were stained for intracellular cytokines. Mean fluorescence intensity (MFI) of TNFα (A), IFNγ (B), IL-17 (C) and IL-10 (D) among CD3 cells (left panels), Vδ1 cells (middle panels) and Vδ2 cells (right panels) from non-pregnant (np, white boxes) and pregnant women (third trimester, 3T, grey boxes) were analyzed. Box plots show the median and the interquartile ranges. *P < 0.05, **P < 0.01, ***P < 0.001. (PDF 55 kb) [file 13075_2016_925_MOESM2_ESM.pdf]
